# Community-based adult hearing care provided by community healthcare workers using mHealth technologies

**DOI:** 10.1080/16549716.2022.2095784

**Published:** 2022-08-12

**Authors:** Caitlin Frisby, Robert H. Eikelboom, Faheema Mahomed-Asmail, Hannah Kuper, Tersia de Kock, Vinaya Manchaiah, De Wet Swanepoel

**Affiliations:** a Department of Speech-Language Pathology and Audiology, University of Pretoria, Pretoria, South Africa; b Virtual Hearing Lab, Collaborative initiative between University of Colorado and the University of Pretoria, Aurora, CO, USA; c Ear Science Institute Australia, Subiaco, WA, Australia; d Ear Sciences Centre, Medical School, The University of Western Australia, Nedlands, WA, Australia; e Faculty of Health Sciences, Curtin University, Bentley, WA, Australia; f International Centre for Evidence in Disability, London School of Hygiene & Tropical Medicine, London, UK; g hearX Foundation, Pretoria, South Africa; h Department of Otolaryngology-Head and Neck Surgery, University of Colorado School of Medicine, Aurora, CO, USA; i UCHealth Hearing and Balance, University of Colorado Hospital, Aurora, CO, USA; j Department of Speech and Hearing, School of Allied Health Sciences, Manipal University, Manipal, India

**Keywords:** Hearing loss, Community-based rehabilitation, community health, mHealth, teleaudiology, low- and middle-income countries

## Abstract

**Background:**

The rising prevalence of hearing loss is a global health concern. Professional hearing services are largely absent within low- and middle-income countries where appropriate skills are lacking. Task-shifting to community healthcare workers (CHWs) supported by mHealth technologies is an important strategy to address the problem.

**Objective:**

To evaluate the feasibility of a community-based rehabilitation model providing hearing aids to adults in low-income communities using CHWs supported by mHealth technologies.

**Method:**

Between September 2020 and October 2021, hearing aid assessments and fittings were implemented for adults aged 18 and above in two low-income communities in the Western Cape, South Africa, using trained CHWs. A quantitative approach with illustrative open-ended questions was utilised to measure and analyse hearing aid outcomes. Data were collected through initial face-to-face interviews, telephone interviews, and face-to-face visits post-fitting. Responses to open-ended questions were analysed using inductive thematic analysis. The International Outcome Inventory – Hearing Aids questionnaire determined standardised hearing aid outcomes.

**Results:**

Of the 152 adults in the community who self-reported hearing difficulties, 148 were successfully tested by CHWs during home visits. Most had normal hearing (39.9%), 24.3% had bilateral sensorineural hearing loss, 20.9% had suspected conductive hearing loss, and 14.9% had unilateral hearing loss, of which 5.4% had suspected conductive loss. Forty adults met the inclusion criteria to be fitted with hearing aids. Nineteen of these were fitted bilaterally. Positive hearing aid outcomes and minimal device handling challenges were reported 45 days post-fitting and were maintained at six months. The majority (73.7%) of participants fitted were still making use of their hearing aids at the six-month follow-up.

**Conclusions:**

Implementing a hearing healthcare service-delivery model facilitated by CHWs in low-income communities is feasible. mHealth technologies used by CHWs can support scalable service-delivery models with the potential for improved access and affordability in low-income settings.

## Background

Hearing loss is a global health concern, with estimates indicating that one in four individuals worldwide will have a disabling hearing loss by 2050 [1,2]. Currently, the prevalence is approximately four times higher in low- and middle-income countries (LMICs) than in higher-income countries [3,4] due to more environmental risk factors and poor hearing healthcare services [2,5,6]. Hearing healthcare services are unavailable to most people globally due to a dearth of hearing care professionals, the prohibitive expense of traditional equipment, and centralised models of care [2,5–8]. LMICs, particularly in areas such as Africa, have fewer than one audiologist per million persons and almost no hearing healthcare infrastructure across most of the region [2].

The most common treatment for hearing loss is hearing aids [9], which are associated with improved health-related quality of life related to communication, socio-emotional and cognitive functioning [2,10,11]. More than 90% of persons with disabling hearing loss on the African continent do not have access to hearing aids [2]. Even if available, hearing aids are often unaffordable, especially in LMICs [12,13]. At the same time, untreated hearing loss is associated with an annual global cost of one trillion USD [2].

The recent World Report on Hearing [2] recommends priorities to improve access and affordability of hearing care. Task-shifting through the training of lay or community healthcare workers (CHWs) is proposed as a primary strategy to deliver hearing healthcare services, especially in LMICs [1,2,14,15]. Task-shifting to CHWs can be supported by mobile health (mHealth) digital technologies designed to offer hearing healthcare services, such as screening, in communities. mHealth tools such as the hearScreen^TM^ and hearTest^TM^ have enabled CHWs to provide hearing screening [16–18] and diagnostic testing comparable to that of professionals [19] through the use of automated testing procedures and quality controls like noise-level monitoring [17–19]. CHWs have also successfully fit adults and children with hearing aids [20,21]. However, there is limited evidence of CHWs providing end-to-end hearing care supported by mHealth technologies [22].

Automated hearing testing with smartphone-based in-situ audiometry conducted through hearing aids can support community-based hearing aid fittings [23,24]. In-situ hearing thresholds have been shown to be accurate, reliable [25,26], and comparable to thresholds obtained via gold standard audiometry testing [27]. Circumaural ear protection placed over hearing aids can reduce ambient noise when testing in homes and primary healthcare settings [28]. In-situ audiometry also enables hearing aid programming directly from a smartphone with no significant differences in hearing outcomes, perceived level of hearing difficulties, and speech recognition compared to conventional hearing aid fittings [29,30]. Advances in hearing aid technology, such as feedback management and non-custom ear moulds, make hearing aids suitable for most degrees of hearing loss [12]. These technologies can support CHWs to improve and simplify hearing healthcare in underserved communities [31–33].

Despite validation research for hearing aid service-delivery components using mHealth technologies, there is minimal evidence for service-delivery models implemented to improve community-based hearing care, including screening through to treatment [34,35]. Implementation science provides a valuable research framework to incorporate evidence-based practice into current standard practice [34]. Implementation science aims to examine several outcomes in audiology research, including acceptability, feasibility, and sustainability [35]. Therefore, this implementation study aimed to evaluate the feasibility of a community-based rehabilitation (CBR) model for hearing assessment and hearing aid provision to adults in low-income communities by CHWs supported by mHealth technologies.

## Method

The Bowen et al. [36] feasibility study framework guides the design of feasibility studies according to eight possible focus areas. One possible focus area is implementation which explores the possibility of implementing an intervention as planned in an uncontrolled manner [36, 37]. This study used an implementation science approach for designing and evaluating the outcome of the CBR-based hearing aid service-delivery model [34]. A quantitative approach with illustrative open-ended questions was utilised to collect and analyse data. Qualitative data are essential when implementation is evaluated in order to explore stakeholder opinions and perspectives [35]. Data were collected through initial face-to-face interviews, telephone interviews, and face-to-face visits post-fitting. Responses to open-ended questions were analysed using inductive thematic analysis. Standardised structured outcome measures were also utilised. The implementation was evaluated to determine the extent to which this service-delivery model could be successfully delivered to low-income communities [36].

### Service-delivery model

**Figure 1.**
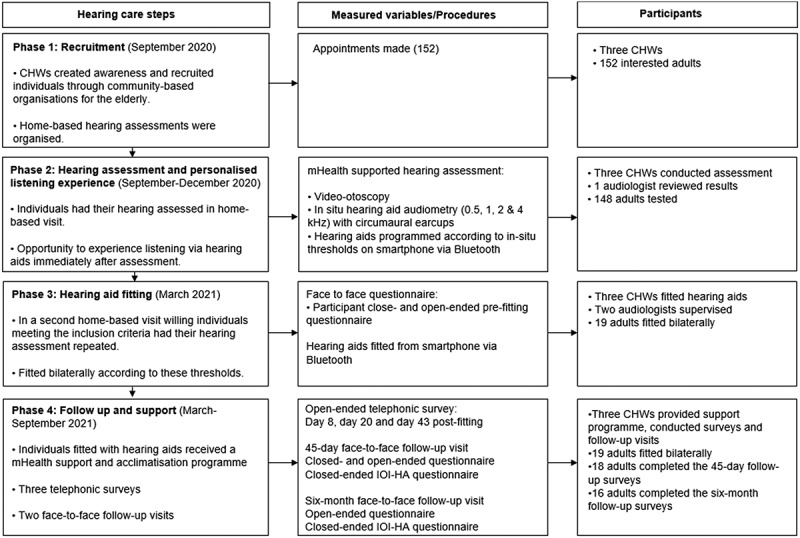
Four phases in the mHealth service-delivery model, using CHWs to implement hearing healthcare in LMICs.

This CBR service-delivery model was implemented and evaluated in two low-income communities (i.e. Khayelitsha and Mbekweni) in the Western Cape, South Africa, in collaboration with a local non-governmental organisation (NGO) (i.e. hearX Foundation). These two low-income communities are located 30 km and 66 km respectively from Cape Town, with estimated populations of 391,749 and 30,875, respectively [38,39]. Khayelitsha has 118,810 households, of which 44.6% are formal dwellings [38]. Mbekweni has 8,339 households, with 63.5% being formal dwellings [39]. The program was facilitated by three CHWs, supervised by two audiologists. Convenience sampling was used to select these communities since the NGO partner (hearX Foundation) has worked with community leaders on community-based projects in the area, and there was a pre-identified need for hearing services. This study was conducted over 13 months, from September 2020 to October 2021. The CBR model comprised four phases, including 1) recruitment, 2) hearing assessment and personalised listening experience, 3) hearing aid fitting, and 4) follow-up and support (Figure 1).

### Phase 1: recruitment

This phase involved recruiting possible participants for the service-delivery model.

#### Training and supervision of CHWs

Purposive sampling was used to recruit three CHWs who were community members and were fluent in Xhosa. The CHWs were previously trained by the NGO partner (hearX Foundation) on hearing screening services for children in the community [17]. Before the training and their two years’ experience in conducting childhood screenings, these CHWs had no previous experience with hearing healthcare.

The NGO partner (hearX Foundation) facilitated further training for the adult service reported in this study through three full-day sessions (08:30–15:00) before data collection commenced. The first session included information on the test protocol, video-otoscopy, in-situ smartphone testing, hearing aid features and functionalities, hearing aid insertion, measurement of tube length, and device maintenance. CHWs then practised on each other and then on the audiologist. The second session covered hands-on practice in the community, where one of the audiologists tested two individuals while the CHWs observed. The CHWs then took turns testing community members’ hearing under the supervision of the audiologist (Figure 1). The final session included training on instructing the participants on use and maintenance of hearing aids.

Two qualified audiologists, the first and fifth author, supervised the program. This included providing training to the CHWs and observing the hearing assessment and hearing aid fitting process.

#### Recruitment of adults

Snowball sampling was used to recruit adults aged ≥ 18 years with self-reported suspected hearing loss. The CHWs contacted community leaders who spread awareness throughout the community through word-of-mouth. Individuals interested in receiving hearing services were given the contact details of the CHWs. CHWs contacted interested individuals and arranged home-based visits once written consent had been obtained. All participants’ home language was Xhosa, and they resided in Khayelitsha (n = 145; 95%) or Mbekweni (n = 7; 5%).

### Phase 2: hearing assessment and personalised listening experience

**Figure 2.**
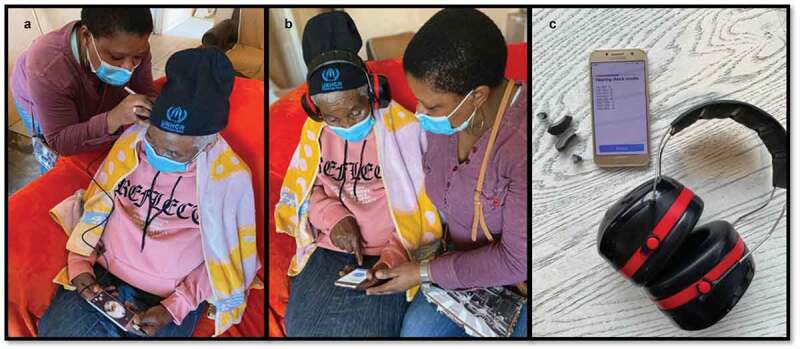
(a): Participant’s ear canal and tympanic membrane being evaluated by a CHW via video-otoscopy. (b): Participant having a hearing assessment via in-situ audiometry and circumaural ear cups. (c): Hearing aids, smartphone, and circumaural ear cups used in service-delivery model.

From September to December 2020, adults with self-reported hearing difficulties who contacted the CHWs to indicate their interest in taking part in this study were visited by the CHWs for the hearing assessments (Figure 2). As far as possible, a quiet space in participants’ homes was used for testing. COVID-19 regulations to minimise the risk of virus transmission were in place. Smartphone video-otoscopy (hearScope^TM^; hearX Group, Pretoria, South Africa) (wired to the smartphone) with artificial intelligence image classification (hearScope^TM^ Beta version) was used to evaluate individuals for possible ear disease, cerumen impaction, and ear canal patency to accommodate a hearing aid. Image classification categorised video-otoscopy results into either normal or abnormal (i.e. possible middle ear pathology or cerumen impaction). If the classification categorised video-otoscopy as abnormal, CHWs made referrals to the local clinic or ear, nose and throat specialist for treatment.

CHWs facilitated hearing assessments using automated in-situ pure tone audiometry (0.5, 1, 2 and 4 kHz) through Lexie Lumen (hearX Group, South Africa) hearing aids. Circumaural Peltor 3 M ear protectors covered the hearing aids to reduce ambient noise levels during testing. These hearing aids produce pure tones for audiometry when connected via Bluetooth to a smartphone application. Responses were recorded by pressing the response button on the smartphone’s screen. A new set of tulip domes was coupled to the hearing aids with slim tubes sized according to each participant’s ear canal size.

Hearing loss results were classified into bilateral or unilateral sensorineural hearing loss (SNHL) (pure tone average ≥ 26 decibels hearing level (dB HL) [40]) when there were no obvious signs of possible ear disease (e.g. cerumen impaction, perforation, evidence of discharge) as determined via smartphone video-otoscopy and image classification. Suspected bilateral or unilateral conductive hearing loss was determined through obvious signs of possible ear disease via smartphone video-otoscopy and image classification. If the participant’s hearing thresholds exceeded 85 dB HL (i.e. maximum output of hearing aids), relevant referrals were made by the CHWs.

After the assessment, hearing aids were immediately programmed according to the participant’s hearing thresholds. This allowed for a personalised listening experience via the hearing aid according to their degree and configuration of hearing loss.

### Phase 3: hearing aid fitting

Hearing aid fittings took place in March 2021. Inclusion criteria to be considered for hearing aid fitting included: i) being ≥ 18 years; ii) bilateral hearing loss (pure tone average ≥ 26 dB HL [40]) with thresholds no greater than 85 dB HL due to maximum output of the hearing aids; iii) normal middle ear functioning determined through visual inspection via a digital otoscope; iv) no previous experience with hearing aids before the first session with CHWs; v) able to receive WhatsApp messages (themselves or a household member); vi) willing and able to purchase batteries monthly beyond the life of the project (approximately 3.4 USD (ZAR50) per pack at pharmacies), and vii) willing to partake in follow-up surveys and interviews.

Individuals who had their hearing tested in Phase 2 and met the inclusion criteria were contacted and asked if they were interested in being fitted bilaterally with hearing aids. A second home visit was arranged if they agreed and were willing to participate in Phase 3. The CHWs facilitated an automated re-test to confirm participants’ hearing ability. A new pair of hearing aids were used to re-test participants’ hearing thresholds and were then fitted bilaterally (Figure 1).

The Lexie Lumen digital hearing aids have 16 channels, wide-dynamic-range compression technology, feedback reduction, Bluetooth connectivity and programming, digital noise reduction, and a directional microphone array. These hearing aids cost less than 180 USD (ZAR2878) per pair. The hearing aids allow for Bluetooth hearing aid fitting, using the NAL/NL2 fitting algorithm from a smartphone application (Lexie Hearing) based on the four thresholds tested.

Immediately after the re-test, participants were fitted with hearing aids. The CHWs facilitated the hearing aid fitting while the audiologists observed. A single touch on the smartphone programmed the aids according to the NAL/NL2 fitting algorithm, based on the four thresholds tested. All participants received their hearing aids free of charge. Participants received a drying kit, extra tubes, domes, and battery packs (enough for the study’s duration) to use at home if replacements were necessary.

Participants were given an open- and close-ended questionnaire (translated to Xhosa) to complete regarding their experiences with hearing loss before the hearing aid fitting. The CHWs completed these questionnaires in an interview format (Supplementary Data I).

### Phase 4: follow-up and support

Participants were contacted by telephone three times over a 45-day period by the CHWs on days 8, 20 and 43 post hearing aid fitting to answer open-ended questions (Supplementary Data I), enquiring about any hearing aid difficulties the participants faced. Additionally, for six weeks, the participants received support in the form of information provided in Xhosa via text message or WhatsApp messaging services (a voice note in Xhosa supplemented by an image in English) (Supplementary Data I). Home-based follow-up visits were conducted by the CHWs independently 45-days and six months post hearing aid fitting. At each follow-up, participants completed open- and close-ended questionnaires (Supplementary Data I) in an interview format. Topics included hearing aid use, difficulties with hearing aids, willingness to pay for hearing aids, treatment from other individuals, concerns, and recommendations. The International Outcome Inventory – Hearing Aids (IOI-HA; Supplementary Data I) was also completed in an interview format at both follow-up visits.

The IOI-HA is used as a self-reported measure of hearing aid effectiveness [41] and covers the topics of daily use, benefit, residual activity limitations, satisfaction, residual participation restrictions, impact on others, and quality of life. There are seven close-ended questions with five possible responses to each question. These responses are arranged from left to right, with the worst outcome with hearing aids on the left scored as one point and the last option on the right being the best outcome with the hearing aids scored as five points [41]. Researchers have generally used the mean scores to interpret the results of IOI-HA. However, as the IOI-HA results in ordinal data, the use of median scores of each item and total scores for the overall scores may be more appropriate [42]. To ensure backward compatibility of the data with previous literature, we also report the mean IOI-HA scores. All questionnaires were translated to Xhosa according to the prescribed translation process [43,44].

### Data analysis

Raw data were captured on data collection sheets, recorded onto a Microsoft Excel (2016) spreadsheet in a numerical format, and imported for statistical analysis into the program Statistical Package for the Social Sciences (SPSS, v27. Chicago, Illinois). Descriptive statistics, including mean and standard deviations (sd), were determined for participant age, gender, and degree of hearing loss. Descriptive statistics, including mean, sd, median, and interquartile range (IQR) of the IOI-HA, were determined to interpret participant outcomes with hearing aids. Mean scores of the IOI-HA were compared to scores obtained in previous research articles of a similar nature. The Wilcoxon signed-rank test was conducted to determine if differences between the IOI-HA scores at the 45-day and the six-month follow-up visits were statistically significant (*p* < 0.001). Qualitative questionnaires were analysed by the first author using inductive thematic analysis to determine emerging themes. The first author then narrowed identified themes down into general themes. The last author then reviewed these themes. Disagreements were discussed and resolved. Testimonials were recorded as participant quotes.

## Results

### Phase 1: recruitment

A total of 152 adults contacted the CHWs, indicating interest in having their hearing assessed. The majority (n = 117; 77%) of these 152 adults were female. One of the participants did not disclose their age, but the remaining 151 ranged from 18 to 102 years of age (mean 59.1; sd 14.9).

### Phase 2: hearing assessment and personalised listening experience

CHWs successfully tested 148 of the 152 adults in the community during home visits over four months (September to December 2020). The 148 adults ranged from 18 to 102 years of age (Mean 58.7; sd 15.3), and 113 (76.4%) were female. Most of the participants had normal hearing (n = 59; 39.9%), 36 (24.3%) had sensorineural hearing loss bilaterally, 31 (20.9%) had suspected conductive hearing loss as determined through video-otoscopy, and 22 (14.9%) had unilateral hearing loss of which eight (5.4%) had suspected conductive loss. Two of the adults presenting with suspected conductive hearing loss bilaterally had discharging ears which prohibited determining hearing thresholds. Hearing thresholds of 146 adults were therefore determined (Table 1).

**Table 1.** Participant demographics and hearing threshold results for those assessed (n = 146) and those subsequently fitted with hearing aids (n = 19).

	Adults screened n = 146	Adults fitted with hearing aids n = 19
**Age (years)**		
Mean (sd)	59.3 (14.8)	71.7 (13)
Range	18–102	48–96
**Age distribution (years)**	**% (n)**	**% (n)**
18–39	7.5 (11)	n/a
40–59	43.2 (63)	21.1 (4)
60–79	41.1 (60)	52.6 (10)
80+	8.2 (12)	26.3 (5)
**Sex**	**% (n)**	**% (n)**
Male	23.3 (34)	21.1 (4)
Female	76.7 (112)	78.9 (15)
**Hearing (0.5, 1, 2 and 4 kHz pure tone average)**		
Mean four frequency PTA (sd) Left	30 (20)	44.2 (13.4)
Mean four frequency PTA (sd) Right	29.1 (18.5)	46.4 (12.9)
PTA Range Left	10–80	26.3–73.8
PTA Range Right	10–80	25–67.5
**Degree of hearing loss (better ear PTA)**	**% (n)**	**% (n)**
Normal (0–24 dB HL)	62.3 (91)	n/a
Mild (25–40 dB HL)	22.6 (33)	42.1 (8)
Moderate (41–60 dB HL)	10.3 (15)	47.4 (9)
Moderately Severe (61–80 dB HL)	4.8 (7)	10.5 (2)
Severe (81+ dB HL)	n/a	n/a
**Period of self-reported hearing loss (years)**	**% (n)**	**% (n)**
1–5	n/a	57.9 (11)
6–10	n/a	5.3 (1)
11–20	n/a	26.3 (5)
20+	n/a	10.5 (2)

### Phase 3: hearing aid fitting

**Figure 3.**
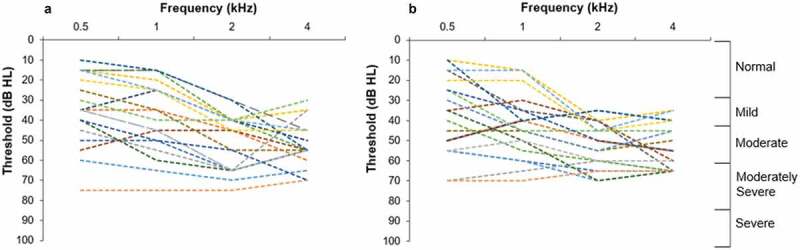
Individual hearing thresholds for participants (n = 19) fit bilaterally with hearing aids according to degree of hearing loss. (a): Left ear. (b): Right ear.

Of the 146 individuals tested, 40 met the inclusion criteria for bilateral hearing aids. Of these, 28 agreed to participate in the hearing aid fitting phase. During this phase (two-week period in March 2021), 19 of these 28 could be contacted and agreed to be fitted with hearing aids. The CHWs re-tested and fitted these 19 individuals with hearing aids bilaterally. Participants’ characteristics are summarised in Table 1, and hearing thresholds are illustrated in Figure 3.

Most (n = 16; 84%) participants indicated that they *felt excited* to try hearing aids, two participants (11%) selected feeling *OK about wearing a hearing aid*, and one (5%) indicated that they were *scared to wear a hearing aid*. Most (n = 9; 47%) participants had never observed another person wearing hearing aids, while eight (42%) participants reported knowing someone with hearing aids. Only two participants reported observing strangers wearing hearing aids. The qualitative perceptions reported about their hearing and use of hearing aids before hearing aid fitting are summarised in Table 2.

**Table 2.** Inductive thematic analysis of participant (n = 19) responses to open-ended questions on awareness of hearing aids and perceptions of hearing loss before hearing aid fitting.

Themes	Frequency	Example quotes
**How has your hearing problem affected your life?**		
Difficulty in conversations	11	*‘I can’t communicate properly with my family members.’*
Difficulty with phone calls	3	*‘I can’t hear on calls; I always ask for repetition.’*
Difficulty in church	2	*‘It is difficult to hear at church’ ‘I am not able to hear at church.’*
Causes worry or embarrassment	2	*‘I feel worried and embarrassed’ ‘Sometimes people laugh at me.’*
Not affected	1	*‘It has not affected my daily life.’*
**How do other people treat you because of your hearing problem?**		
Normal	8	*‘No one has noticed that I cannot hear properly’ ‘They treat me normally.’*
Supportive and understanding	6	*‘They are very understanding’ ‘My family is very supportive.’*
With irritation	5	*‘They get irritated as they have to repeat all the time’ ‘They ignore me most of the time.’*

### Phase 4: follow-up and support

#### Phone calls

Participants were contacted telephonically by the CHWs on days 8, 20 and 43 post fitting to enquire about any possible challenges. Over this period, only four participants reported difficulties: feedback from the hearing aid, confusion regarding drying kit, hearing aid falling out of the ear, and hearing aids no longer working. During home visits, the CHWs discovered the cause of feedback in one individual to be a headscarf blocking hearing aid and that the provided tulip dome was too large. CHWs successfully replaced the dome, counselled the participant, and taught one participant about drying kit usage. They counselled another participant on the correct placement of the hearing aids, advising them to use a mirror when placing the hearing aids in the ear. The last participant reported that the hearing aids were no longer working. The CHWs determined that the batteries were stored in the drying kit and drained rapidly as a result.

#### Follow-up – 45 days and six months

CHWs conducted home visits 45 days and six months post fitting. By day 45, one participant dropped out of the study (refused to continue). Two further participants dropped out between the 45-day and six-month follow-up (one refused, one lost hearing aids). Most of the remainder (n = 14/16; 87.5%) reported that they were still using their hearing aids. Two (12.5%) no longer used their hearing aids (one owing to theft, one owing to pain). Upon inspection by the CHW, the pain was found to be because the participant incorrectly placed a left tube on the right hearing aid. The CHWs successfully replaced the incorrect tube and counselled the participant on correct tube replacement.

When asked about willingness to pay for hearing aids at both follow-up visits, all participants indicated willingness. Amounts ranged from 3.3 to 129.9 USD (ZAR50 to ZAR2000) as an immediate full payment or 3.3 to 32.5 USD (ZAR50 to ZAR500) per month. When asked about difficulties experienced with the hearing aids, 10 (56.6%) participants experienced no difficulties. In comparison, 4 (22.2%), 3 (16.7%), and 1 (5.6%) participants reported difficulties with putting the hearing aid on, cleaning, and putting in new batteries, respectively. Table 3 includes participants’ perceptions of how others have treated them since they obtained their hearing aids.

**Table 3.** Inductive thematic analysis of participant (n = 18) responses to an open-ended question 45 days post fitting on perceptions of others’ treatment towards them since hearing aid fitting.

Themes	Frequency	Example quotes
**Did people treat you any differently since you have a hearing aid?**		
Positively	5	*‘People respond positively to the fact that I wear HAs. They notice that I respond more and quicker than before. They previously thought that I was unfriendly. They did not realise that I could not hear.’*
Same as before hearing aids	9	*‘No. No one has even noticed the hearing aids.’*
With curiosity/interest	4	*‘Yes. People in the street would notice and ask about the hearing aids. Many did not know what it was, so I used it as an opportunity to educate on what it is and why I wear them.’*

All participants completed the IOI-HA questionnaire during the 45-day (n = 18) and six-month (n = 16) follow-up visit (Table 4). At the 45-day follow-up, two participants (11.1%) indicated that they wore their devices one to four hours per day, seven participants (38.9%) wore their devices four to eight hours daily, and nine participants (50%) wore their devices on more than eight hours per day. During the six-month follow-up, seven participants (43.8%) wore their devices one to four hours per day, and six participants (37.5%) wore their devices four to eight hours daily. Three participants (18.8%) wore their devices more than eight hours daily. In the 45-day follow-up, 17 (94.4%) participants reported that the hearing aids helped them to hear better in different situations, while only one (5.6%) participant who presented with a mild to moderate hearing loss felt the hearing aids did not help. During the six-month follow-up, all 16 participants felt the hearing aids helped them to hear better in different situations. These results indicate that most participants experienced positive outcomes with their hearing aids. The difference in average scores across the IOI-HA categories at the 45-day and the six-month follow-up visit ranged from −0.2 to 0.6. IOI-HA scores at the 45-day and the six-month follow-up visits, and were not statistically significant across all questions (p > 0.05; Wilcoxon signed-rank test).

**Table 4.** IOI-HA median, IQR, mean, and sd scores at the 45-day (n = 18) and six-month (n = 16) follow-up.

	45-day follow-up		Six-month follow-up	
	Median (IQR)	Mean (sd)	Median (IQR)	Mean (sd)
Daily Use	4.5 (4–5)	4.4 (0.7)	4 (3–4)	3.8 (0.8)
Benefit	5 (5–5)	4.6 (1)	5 (5–5)	4.8 (0.4)
Residual Activity Limitation	5 (4.8–5)	4.6 (1)	5 (5–5)	4.8 (0.6)
Satisfaction	5 (4–5)	4.6 (0.5)	5 (5–5)	4.8 (0.4)
Residual Participation Restrictions	5 (5–5)	4.9 (0.5)	5 (4–5)	4.7 (0.5)
Impact on others	5 (4–5)	4.6 (0.6)	5 (5–5)	4.3 (1.5)
Quality of Life	5 (4.8–5)	4.7 (0.6)	5 (4.3–5)	4.7 (0.6)

Table 5 reports participant responses to open-ended questions covering the impact of hearing aids on their lives, difficulties and recommendations. Of the original 19 participants, 14 (73.7%) were still using their hearing aids at the six-month follow-up.

**Table 5.** Inductive thematic analysis of participant (n = 16) responses on impact of hearing aids on their lives, difficulties, and recommendations at six-month follow-up.

Themes	Frequency	Example Quotes
**If you still use your hearing aids, can you share how the hearing aids have impacted your life?**		
Improved hearing over phone	3	*‘My life has been changed a lot. I am able to hear the phone better now.’ ‘Can hear better now over the phone during phone calls.’ ‘I can speak on the phone freely.’*
Improved hearing at church	4	*‘I normally wear them to church and can now hear the pastor well.’ ‘Even at church, I can hear the pastor better.’ ‘I can hear the pastor nicely now at church.’*
Improved environmental sounds	4	*‘I can hear cars on the road when I am going to town.’ ‘When I go to town, I can hear the sounds of the cars and the people at the shops.’ ‘Helps a lot at the malls.’*
Improved communication	6	*‘I can hear my family nicely now when we are talking.’ ‘Around the home, I can engage with the family and participate in any family discussions.’ ‘Helps when talking to people.’*
Improved TV/Radio enjoyment	4	*‘Helping a lot, especially when watching TV.’ ‘It has changed my life; I can hear very well from far, and I don’t need the radio to be as loud anymore.’ ‘Everything is fine now; my life has been changed. No need to turn up the TV or radio volume anymore.’*
No longer using hearing aids	2	*n/a*
**If still using your hearing aids how often do you wear your hearing aids?**		
Daily	9	*‘I wear them every day without being helped.’*
A few days a week	1	*‘Couple of days a week.’*
Twice a week	1	*‘Twice a week’*
Three to four times a week	2	*‘3 to 4 times a week.’*
Only for certain events	1	*‘Only for certain events like going to the shops.’*
Not applicable	2	*n/a*
**Are you experiencing any difficulties with your hearing aids?**		
No	14	*‘No difficulties at all, everything is good’ ‘I am not experiencing any difficulties. They fit me nicely.’*
Pain	1	*‘Pain mostly in the right ear.’*
Discomfort	1	*‘When I put the hearing aids on, it does not feel comfortable, like it shocks or shivers, but the volume is not too loud.’*
**Are you able to change the batteries?**		
Able to change batteries Independently	12	*‘I am able to change batteries myself whenever they are flat.’*
Family member assists	4	*‘Grandchildren and daughter help change the batteries whenever it is necessary.’*
**Are you able to clean your hearing aids?**		
Able to clean the hearing aids independently	10	*‘I am able to clean the hearing aids myself at the end of the day.’*
Family member assists	5	*‘I clean them twice a week with the help of my son.’*
Not cleaned	1	*‘They have not been cleaned. My grandchild does not help me as he is supposed to do.’*
**Do you have any concerns about wearing your hearing aids?**		
No	14	*‘No concerns, the hearing aids made a big difference.’*
Pain	1	*‘The right ear is sometimes painful.’*
Discomfort	1	*‘No concerns except the shivering/shock I experience when putting on the hearing aids or shortly after.’*
**Would you recommend hearing aids to other people with hearing difficulties? Why/Why not?**		
Yes, they help to hear better	6	*‘Yes, because it improves hearing’ ‘Yes, I would recommend it because it helps to hear, and it can help someone else too.’*
Yes, they allow for better communication	5	*‘Yes, they allow you to talk nicely with your family’ ‘I would recommend the hearing aids as a good tool to hear well, and more people will feel free to communicate with you if you have hearing aids because they won’t have to repeat.’*
Yes, they are very helpful	5	*‘Yes, I recommend it a lot as hearing aids are very helpful’ ‘Yes, the hearing aids help a lot. They change lives.’*
**What advice would you give to someone who is struggling to hear?**		
Go to clinic/doctor for hearing test	9	*‘Go to the clinic to have their hearing tested’ ‘I would advise them to visit the clinic or doctor to get them the help they need.’*
Get hearing aids	3	*‘Advise them to go and get hearing aids’ ‘I would advise them to buy hearing aids.’*
Contact CHWs	9	*‘I will give him/her the advice to look for the CHWs.’ ‘Contact the CHWs to help.’*

## Discussion

This implementation study evaluated the feasibility of a CBR model providing hearing aids to adults in low-income communities using CHWs supported by mHealth technologies. With appropriate training, CHWs recruited participants, facilitated hearing assessments, made referrals, conducted hearing aid fittings, and provided post-fitting support using mHealth technologies. According to Bowen’s criteria, this CBR model was in demand, acceptable, and practical to implement. This was illustrated through community members’ expressed interest, actual use of hearing aids by participants, and participant satisfaction with services [36]. Furthermore, this model allowed for health equity where a disparity in hearing healthcare currently exists [35]. This CBR model demonstrates that decentralised hearing healthcare services by CHWs in low-income communities are possible using mHealth technologies.

The World Health Organization identifies task-sharing to CHWs as a key priority in providing community-based hearing healthcare services [2]. Using this approach, CHWs have successfully provided a range of hearing healthcare services, including screening and triage of at-risk individuals [14–16,19]. Community-based hearing assessments and hearing aid fittings outcomes also compare well to traditional clinic-based services [20]. In addition to successfully assessing hearing loss, the CHWs in this study successfully fitted participants bilaterally. Studies conducted in India and Bangladesh also concluded that, if trained, CHWs could successfully fit adults and children with conventional hearing aids in a community setting [20,21]. Employing mHealth tools used by CHWs to facilitate hearing testing, hearing aid fitting, and support made this a unique study of a comprehensive community-based model for adult hearing care.

Implementing this service-delivery model in a community setting through CHWs enabled home-based visits. Although not investigated in this study, a benefit of home-based services is that the time and costs associated with travelling to clinics could potentially be eliminated [20]. The risk of contracting COVID-19 by participants who are usually elderly and most at risk was also reduced as home-based visits were conducted in less crowded and better ventilated environments than traditional hearing healthcare clinics [45,46]. Furthermore, the CHWs were also members of these communities, and all instructions and information could be provided in the participants’ home language. Healthcare provided in a language that participants are not fully competent in could lead to decreased quality of care [47].

The type of mHealth technologies implemented in this service-delivery model presented several possible benefits, which may have contributed to the successful implementation of this model. The lower costs associated with the hearing aids [25] allowed for hearing healthcare services to be offered in two low-income communities where hearing services are limited. The portability of the hearing aids [48] and the circumaural ear protectors reducing ambient noise made accessibility of the service-delivery model possible. Participants could, therefore, be assessed and fitted during home visits. Participants reported satisfaction with the hearing aids, and 73.7% (n = 14 of 19) of them were still using their hearing aids at the six-month follow-up. Reasons for non-use included two participants no longer willing to participate. One participant lost the hearing aids, another participant’s hearing aids were stolen, and the final participant no longer wore the hearing aids due to pain.

The portability, reduced equipment costs, and digitally inclusive nature of the mHealth technologies allowed the CHWs to easily facilitate the model after minimal training, supporting the scalability of this model. Innovative finance options should be explored to expand this service-delivery model to other LMICs. Participants could, for example, make regular payments through a subscription base, once-off pre-payment before receiving hearing aids, or a payment option via a smartphone application. Although this study did not require participant payments, all participants indicated a willingness to pay between 3.5 USD and 34.7 USD (ZAR50 to ZAR500) monthly. Approximately 74% of households in these low-income communities receive a monthly income of 221.8 USD (ZAR3200) or less [38]. This willingness to pay for hearing aids thus illustrated participant satisfaction and the potential to implement this service-delivery model on a larger scale.

Self-reported hearing aid outcomes (IOI-HA questionnaire) were comparable to those from previous studies in both high-income countries and LMICs, including the USA [41], Germany [49], Philippines [50], and South Africa [51]. These outcomes persisted beyond six months post fitting. Several open-ended questions probed benefit across different listening situations not covered by the IOI-HA [52] post fitting (45 days and six months). Qualitative information is a valuable part of the validation process to acquire information regarding the perceptions of the individuals fitted with the hearing aids [53]. All participants reported improvements in various aspects of their lives owing to their hearing aids. These improvements included hearing over the phone, hearing at church, awareness of environmental sounds, communication, and TV/radio enjoyment. Several participants reported experiencing irritation from other people regarding their hearing loss before the hearing aid fitting, whilst post fitting, no negative perceptions from others were reported. Stigma has been identified as a contributing barrier to hearing aid uptake [2,54], as hearing aids are often perceived as a sign of ageing, disability, or difficulties communicating [54]. The positive experiences of the participants’ post fitting indicate that a community-based CHW service-delivery model could help address the stigma associated with hearing loss and hearing aids.

The success of this service-delivery model highlights the potential generalisability across similar communities in LMICs. However, some potential barriers to future uptake include inability to afford hearing aids in low-income communities. A sustainable service-delivery model would require a form of payment from participants to cover running costs. Furthermore, the high incidence of suspected conductive components in these communities is a possible barrier as healthcare professionals may not be readily available to assist with wax removal or medical treatment.

## Limitations

Qualitative data regarding participant perceptions of the service delivery model were only collected for the sample of participants fitted with hearing aids and not those who only received hearing tests. The majority (n = 15; 78.9%) of participants fitted were females. This may indicate that females are more willing to disclose and seek help for suspected hearing loss or less likely to be working away from the family home. This service-delivery model was implemented during the COVID-19 pandemic. Thus, some individuals may have opted not to contact the CHWs for home-based visits based on concerns about possible COVID-19 infection. This may restrict generalisability. A formalised implementation science framework, such as Bowen’s framework, should be utilised in further research to examine cost-effectiveness compared to audiologist-led fitting. A quantitative approach with illustrative open-ended questions was used in this study. Therefore, further exploration using other analysis methods and other aspects of Bowen’s framework would be essential to support a more holistic and in-depth understanding of the feasibility of this service-delivery model. No verification tools such as real-ear measurements could be conducted to verify the fitting or determine the appropriateness of tulip dome selection.

## Conclusions

An innovative hearing healthcare service-delivery model in low-income communities facilitated by CHWs supported by mHealth technologies is feasible. Using mHealth technologies, CHWs can support scalable service-delivery models with the potential to improve access and affordability in LMICs to manage hearing loss. Participants in this study reported good hearing aid benefit, although controlled studies are necessary to compare this method to standard audiologist-led service-delivery models. Further research should be conducted on the implementation of this service-delivery model in various LMICs to determine the generalisability and scalability of such a model.

## Supplementary Material

Supplemental Material

## Paper context

Task-shifting supported by mHealth technologies has enabled community healthcare workers to provide various hearing healthcare services. Currently, however, there is a lack of evidence for a comprehensive community-based model for adult hearing care. This study evaluated an innovative hearing service-delivery model in a low-income community, using community healthcare workers supported by mHealth technologies. This model has the potential to improve accessibility and affordability in low-income countries to manage hearing loss.
