# Supplementary material for: Community-based adult hearing care provided by community healthcare workers using mHealth technologies
Source: Glob Health Action. 2022 Aug 12;15(1):2095784. doi: 10.1080/16549716.2022.2095784 (PMC9377258; doi:10.1080/16549716.2022.2095784)
Supplement: Supplemental Material [file ZGHA_A_2095784_SM3243.docx]

| **Questions for hearing aid users pre-fitting** | Participant number: |
| --- | --- |

1. How do you feel about wearing a hearing aid?

a) I feel excited.

b)   I feel ok to wear a hearing aid.

c) I feel scared.

d) I feel ashamed or embarrassed.

1. Do you know of someone who wears a hearing aid?
2. Yes     If yes, who?......................................................
3. No

3. For how many years have you had a problem with your hearing?

__________________________________________________________________________________________________________________________________________________________________

4. How has your hearing problem affected your life?

__________________________________________________________________________________________________________________________________________________________________

5. How do other people treat you because of your hearing problem?

__________________________________________________________________________________________________________________________________________________________________

6. How comfortable are you in using a phone?

a) I can receive phone calls.

b) I know how to make and receive calls.

c) I can make and receive calls and send and receive text messages via WhatsApp.

d) I need someone at home to help me in using the phone.

**Script for phone calls**

**Phone Call One: 8 Days after hearing aid fitting**

Hello. My name is........ I am calling from the hearing aid support programme. Well done on wearing your hearing aids for a week! I am calling to find out how it is going? Is this a good time to talk?

- Did you have any difficulty wearing the hearing aids? If yes, what did you struggle with?
- Are you managing to put the hearing aids into your ears? (Or with support from family members?)
- Do you understand how to clean the hearing aids?
- Did you understand the WhatsApp / SMS messages?
- How do the hearing aids sound? (Is it too loud? Are you happy with it?)
- The hearing aid may sound uncomfortable at first because your brain is hearing sounds that it has not heard in a long time. The first part of getting used to it is the hardest but as you wear it more often, the sound will be more comfortable.

**Phone Call Two: 20 days after hearing aid fitting**

Well done on wearing your hearing aids for three weeks! You have now learnt about common problems that happen with hearing aids, and how it can be fixed.

- Have you experienced any problems with your hearing aids?
- Are you getting used to how the hearing aids sound?
- Can you clean the hearing aid?
- Have you heard the "battery low - replace battery" message? Did you put in new batteries? [By now they should have changed the batteries]

**Phone Call Three: 43 days after hearing aid fitting**

Hello! I am (name), calling from the hearing aid support programme. It has been more than 40 days since you got your hearing aids. I am calling to find out how it is going and to hear if you were happy with all the information.

- Do you feel comfortable wearing your hearing aid?
- Are you managing to clean the hearing aid?
- Are you having any problems with wax or dirt in the dome or tube? (The hearing aid will sound blocked or funny.)
- Are you finding anything difficult with your hearing aid? (for example: difficult to change batteries, difficult to hear over the phone?)
- Is there anything you need help with for your hearing aid?
- How often are you wearing your hearing aid?
- I would like to know how this programme was for you. Do you feel like the messages, calls, and pictures have helped you get used to your hearing aid? If not, why? What could we have done better?
- Can we come for a final home visit to check how it is going with the hearing aid and ask some more questions?

**Example of Support Programme Images**


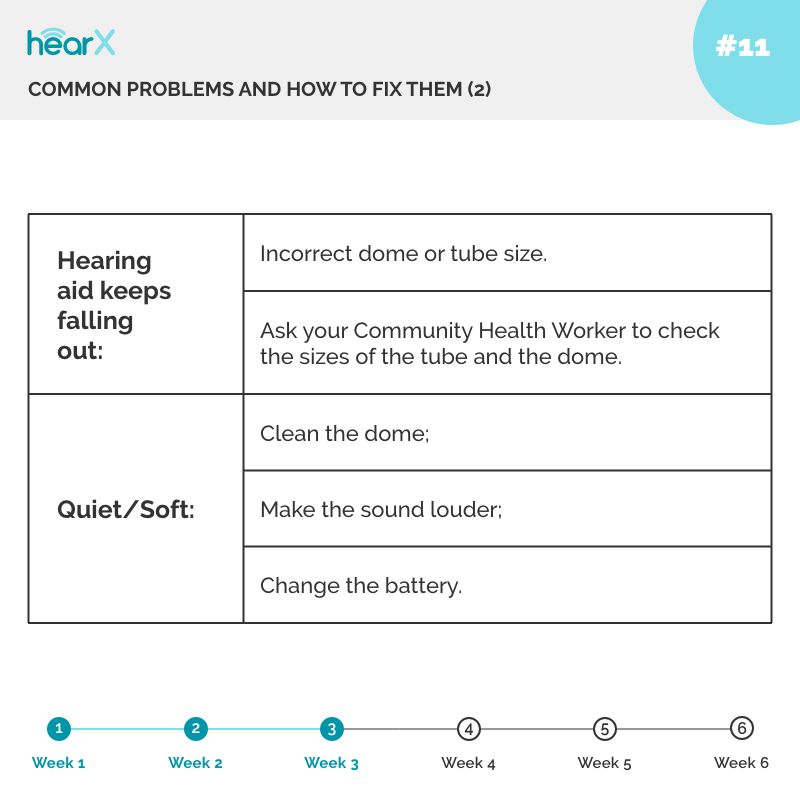

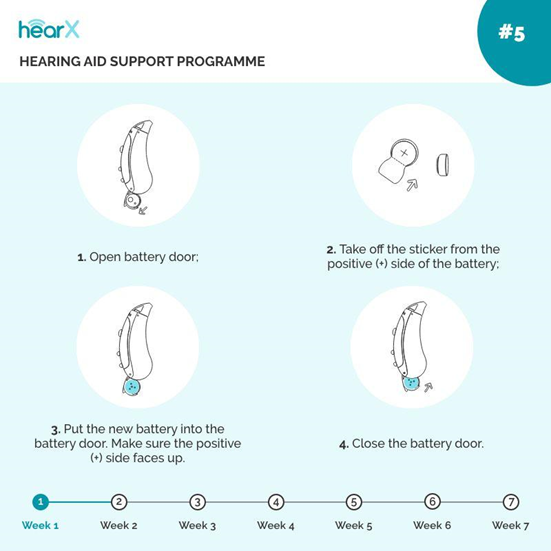
**
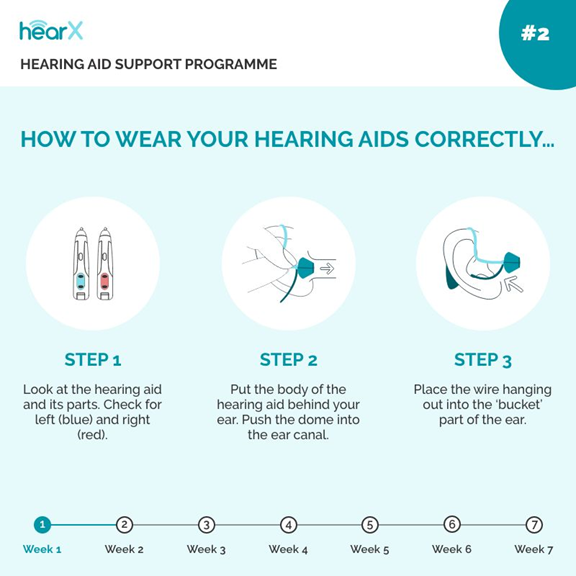
**


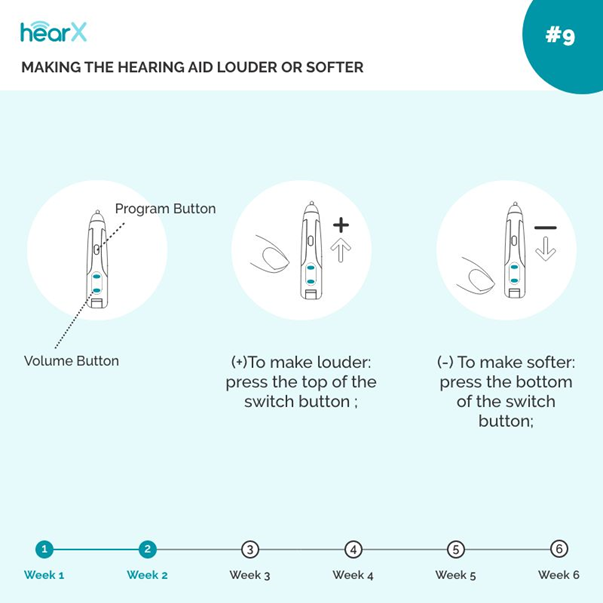


| **Questions for hearing aid users 45 days post-fitting** | Participant number: |
| --- | --- |

General comments: E.g., Were the participants wearing the hearing aid when you (CHWs) arrived

__________________________________________________________________________________________________________________________________________________________________

1. Which of the cases best describe how you feel about your hearing at the moment?

a. I do not think I have a hearing problem, and therefore, nothing should be done about it.

b. I think I have a hearing problem. However, I am not yet ready to take any action to solve the problem, but I might do so in the future.

c. I know I have a hearing problem, and I intend to take action to solve it soon.

d. I know I have a hearing problem, and I am here to take action to solve it now.

2.  Did you find it difficult to perform any of the following tasks:

a. Putting the hearing aid on

b. Cleaning the hearing aid

c. Putting in new batteries

d. Storing the hearing aid

3.  Did people treat you differently after you started wearing a hearing aid?

__________________________________________________________________________________________________________________________________________________________________

4. You can keep your hearing aids free of charge but if you had to pay for a hearing aid would you be willing to pay for the hearing aid? If you are willing to pay for a hearing aid, how much would you be willing to spend?

__________________________________________________________________________________________________________________________________________________________________

General comments: E.g., Needed a new dome for better comfort, needed a different tube size.

__________________________________________________________________________________________________________________________________________________________________

| **6 Month follow-up Questions** | **Participant number:** |
| --- | --- |

1. Are you still using your hearing aids?    YES / NO
2. If YES above - Can you share how the hearing aids have impacted your life?? (e.g., Hear the pastor better in church, can hear better over the phone during phone calls, can hear cars better when going for a walk etc.)

___________________________________________________________________________________________________________________________________________________________________________________________________________________________________________________

1. If YES - how often do you wear your hearing aids? (I.e., every day, couple of days per week, once a week, only for certain events)

___________________________________________________________________________________________________________________________________________________________________________________________________________________________________________________

1. IF NO above - Can you share why you are not using your hearing aids anymore??

___________________________________________________________________________________________________________________________________________________________________________________________________________________________________________________

1. Are you experiencing any difficulties with your hearing aids and if so please explain?

___________________________________________________________________________________________________________________________________________________________________________________________________________________________________________________

1. Are you managing to change the batteries? How often do you change your batteries?

___________________________________________________________________________________________________________________________________________________________________________________________________________________________________________________

7. Have you changed the tubes/domes?

__________________________________________________________________________________________________________________________________________________________________

1. Are you managing with cleaning the hearing aids? How often do you clean your hearing aids? Who cleans it?

__________________________________________________________________________________________________________________________________________________­­­­­­­­­­­­­­­­­­­­________________

1. Do you have any concerns about wearing your hearing aids?

___________________________________________________________________________________________________________________________________________________________________________________________________________________________________________________

1. Would you recommend hearing aids to other people with hearing difficulties? Why/Why not?

____________________________________________________________________________________________________________________________________________________________________________________________________________________________________________________________________________________________________________________________________

1. You can keep your hearing aids free of charge but if you had to pay for a hearing aid would you be willing to pay for the hearing aid? If you are willing to pay for a hearing aid, how much would you be willing to spend?

___________________________________________________________________________________________________________________________________________________________________________________________________________________________________________________

12. What advice would you give to someone who is struggling to hear?

___________________________________________________________________________________________________________________________________________________________________________________________________________________________________________________

General comments (e.g., Were they wearing the hearing aids when you arrived? Did you help them with anything? Any comments by their family / friends regarding the impact of the hearing aids on their life?):

____________________________________________________________________________________________________________________________________________________________________________________________________________________________________________________________________________________________________________________________________

**International Outcome Inventory for Hearing Aids (IOI-HA)**

1. Think about how much you used your present hearing aid(s) over the past two weeks. On an average day, how many hours did you use the hearing aid(s)?

| none | less than 1 hour a day | 1 to 4 hours a day | 4 to 8 hours a day | more than 8  hours a day |
| --- | --- | --- | --- | --- |

1. Think about the situation where you most wanted to hear better, before you got your present hearing aid(s). Over the past two weeks, how much has the hearing aid helped in those situations?

| helped not at all | helped slightly | helped moderately | helped quite a lot | helped very much |
| --- | --- | --- | --- | --- |

1. Think again about the situation where you most wanted to hear better. When you use your present hearing aid(s), how much difficulty do you STILL have in that situation?

| very much difficulty | quite a lot of difficulty | moderate difficulty | slight difficulty | no difficulty |
| --- | --- | --- | --- | --- |

1. Considering everything, do you think your present hearing aid(s) is worth the trouble?

| not at all worth it | slightly worth it | moderately worth it | quite a lot worth it | very much worth it |
| --- | --- | --- | --- | --- |

1. Over the past two weeks, with your present hearing aid(s), how much have your hearing difficulties affected the things you can do?

| affected very much | affected quite a lot | affected moderately | affected slightly | affected not at all |
| --- | --- | --- | --- | --- |

1. Over the past two weeks, with your present hearing aid(s), how much do you think other people were bothered by your hearing difficulties?

| bothered very much | bothered quite a lot | bothered moderately | bothered slightly | bothered not at all |
| --- | --- | --- | --- | --- |

1. Considering everything, how much has your present hearing aid(s) changed your enjoyment of life?

| worse | no change | slightly better | quite a lot better | Very much better |
| --- | --- | --- | --- | --- |

1. How much hearing difficulty do you have when you are not wearing a hearing aid?

| severe | moderately  -severe | moderate | mild | none |
| --- | --- | --- | --- | --- |

Norms for the IOI-HA

Cox, Alexander, & Beyer, 2002

| **Item** | **Individual clients** | | **Groups of clients** | |
| --- | --- | --- | --- | --- |
|  | *Mild-moderate lower/upper* | *Mod-severe+ lower/upper* | *Mild-moderate mean/SD* | *Mod-severe+ mean/SD* |
| *1. use* | 3/5 | 4/5 | 3.73/1.17 | 4.5/.96 |
| *2. benefit* | 3/4 | 3/4 | 3.39/.98 | 3.52/1.08 |
| *3. RAL* | 3/4 | 2/4 | 3.4/.95 | 3.19/1.05 |
| *4. satisfac.* | 2/4 | 3/5 | 3.2/1.21 | 3.84/1.17 |
| *5.RPR* | 3/4 | 3/4 | 3.57/1.13 | 3.38/1.11 |
| *6.imp-oth* | 3/5 | 2/4 | 3.79/1.13 | 3.38/1.1 |
| *7. QofLife* | 3/4 | 3/4 | 3.19/.93 | 3.68/1.02 |

The category of norms used should depend on the patient’s answer to the 8^th^ item of the questionnaire. If they choose “none”. “mild” or “moderate”, use the “mild/moderate” norms. For the other 2 options, use the “mod/severe” norms.

The norms for individual clients are the middle 50% of the data. Hearing aids were: Single-channel, single-memory, ITE; All bilateral fittings; All compression (any type); standard fitting protocol; Purchased between Aug/00 & Jan/01.

IOI-HA norm template for individual scores.

Cox, Alexander, & Beyer, 2002

5


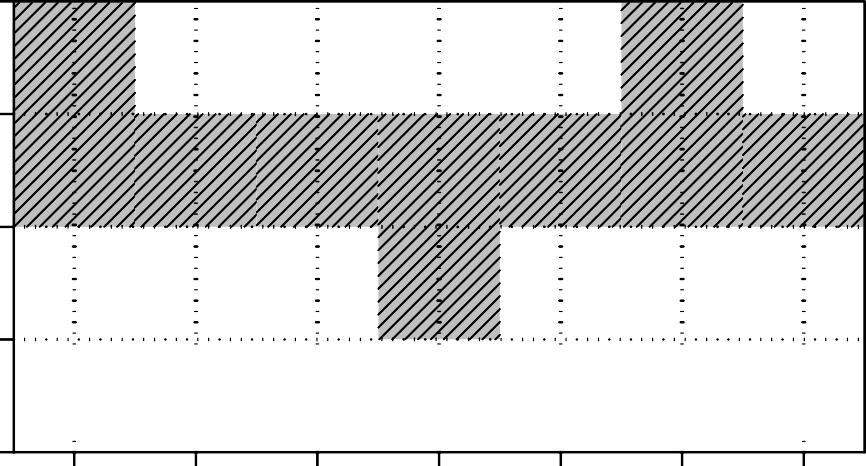

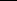

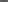

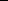

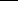

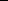

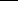

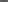

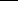

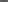

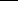

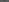

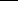

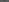

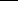

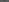

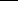

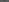

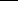

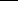

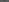

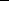

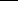

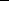

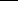

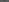

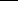


subjective problems = mild-moderate

4

Item Score

3

2

1

Use Ben RAL Sat RPR Ioth QoL

5


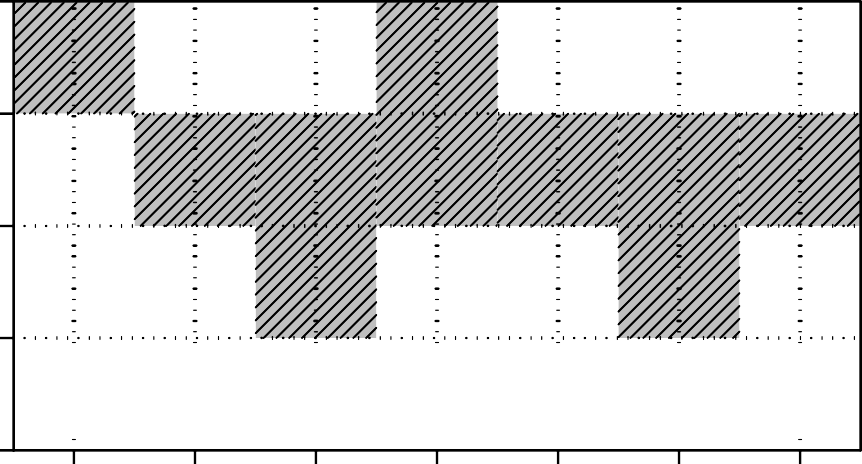

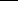

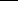

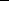

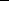

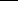

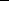

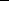

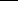

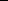

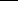

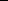

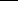

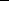

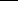

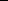

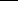

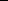

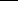

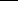

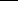

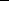

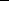

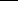

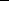

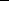

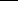


subjective problems = mod-severe+

4

Item Score

3

2

1

Use Ben RAL Sat RPR Ioth QoL
